# Ultrasound reverses chemoresistance in breast cancer stem cell like cells by altering ABCG2 expression

**DOI:** 10.1042/BSR20171137

**Published:** 2017-11-09

**Authors:** Lijuan Guo, Pengfei Zheng, Huijun Fan, Haiyan Wang, Wenzhong Xu, Wenyan Zhou

**Affiliations:** 1 Ultrasonography Department, The First Affiliated Hospital of Xinxiang Medical University, Weihui 453100, Henan Province, People’s Republic of China; 2 Department of Thyroid Breast and Vascular Surgery, The First Affiliated Hospital of Xinxiang Medical University, Weihui 453100, Henan Province, People’s Republic of China

**Keywords:** ALDHA1, ABCG2, breast cancer stem cell-like cells, doxorubicin, microbubble, ultrasound

## Abstract

Doxorubicin (DOX) resistance in breast cancer largely results from the breast cancer stem cell like cells (BCSCs) which could be targetted to improve the efficacy of chemotherapy. Cell permeabilization using microbubbles (MBs) and ultrasound (US) have the potential for delivering molecules into the cytoplasm. We aim to evaluate a new methodology of US on BCSCs. First, our findings indicated that ALDHA1^+^ spheres which were derived from fresh primary breast cancer samples displayed stem cell like features and were resistant to DOX. In patient cohort, we revealed the presence of a variable fraction of ALDHA1^+^cells in nine out of ten. We, for the first time, showed a new US-MB treatment condition which could be used on ALDHA1^+^ BCSCs by fluorescence measurement and calcein assay. Next, we demonstrated the efficacy of combined treatment on human BCSCs *in vitro* and *in vivo* using DOX and US-MB: the combined treatment with much reduced drug dosage significantly suppressed the stem cell like features of BCSCs and induced BCSCs apoptosis. Furthermore, we suggested that decreased ABCG2 level might be one of the mechanisms by which US-MB medicated DOX treatment. In conclusion, this new US-MB treatment condition has clinical potential in breast cancer therapy by targetting BCSCs; thereby holding benefits for breast cancer patients.

## Introduction

Breast cancer is the most common malignancy in women [1]. Resistance to chemotherapeutics is still a major challenge for breast cancer therapy, although early detection methods and treatments have greatly improved survival of breast cancer patients [1]. Nowadays, accumulating evidence have shown that a subpopulation of cancer cells with stem cell like features, including self-renewal, differentiation, tumorigenesis, and tumor heterogeneity. This subpopulation of cancer cells were named cancer stem cell like cells (CSCs) [2]. CSCs are reported as the major source of tumor recurrence after radiation or chemotherapy [3]. Previous studies have reported that CSCs exist in a variety of human malignancies [4,5]. Amongst them, the percentage of breast CSCs (BCSCs) following chemotherapy is increased compared with that before therapy [6]. Residual BCSCs which chemotherapy fails to kill will eventually lead to subsequent disease recurrence in breast cancer patients. Nevertheless, there are still not any strategies helping to remove BCSCs by enhancing their sensitivity to chemotherapy drugs.

Ultrasonic therapy has been used in the cancer treatment for many years but just involving a very limited number of cases. The mechanisms of extracellular molecule uptake into cells involves ultrasound (US) irradiation causing pore formation followed by endocytosis and subsequent cellular repair of the pore in plasma membrane [7]. Ultrasonic irradiation has been proposed as an innovative method for influencing the permeability of cell membranes; therefore facilitating the entry of the related chemotherapy drug into the target cancer cells [8]. Recently, most studies focussed on the low intense US that might have effects on adherent cancer cells, but with very limited antitumor effect. In the meantime, exploration of other intense US, especially highly intense US, is few, in spite of being promising. What is more, there are still no reports focussing on the effect of US on CSCs.

Moreover, it has been demonstrated that US-exposed microbubbles (MB) which have been developed as US contrast agents have a significant additive effect [9]. However, whether US or MB can be used to influence the CSCs’ membranes permeability to make CSCs sensitive to chemoresistance drugs is unknown. Doxorubicin hydrochloride (DOX), an anthracycline antineoplastic agent, has been widely used in solid tumor treatment [10]. Despite its effectiveness, dose-dependent cardiotoxicity is often regarded as the major problem. In the meantime, chemoresistance is the biggest challenge limiting its clinical use. Therefore, heat, irradiation, and US may be potential strategies on the use of DOX. Nevertheless, there are no studies about using these new strategies to make CSCs sensitive to DOX so far.

In the present study, we aim to evaluate a new methodology of US on BCSCs. First, our findings indicated that ALDHA1^+^ spheres which were derived from fresh primary breast cancer samples displayed stem cell like features and were resistant to DOX. In patient cohort, we revealed the presence of a variable fraction of ALDHA1^+^cells in nine out of ten. We, for the first time, showed a new US-MB treatment condition which could be used on ALDHA1^+^ BCSCs by fluorescence measurement and calcein assay. Next, we demonstrated the efficacy of combined treatment on human BCSCs *in vitro* and *in vivo* using DOX and US-MB: the combined treatment with much reduced drug dosage significantly suppressed the stem cell like features of BCSCs and induced BCSCs apoptosis. Furthermore, we suggested that decreased ABCG2 level might be one of the mechanisms by which US-MB medicated DOX treatment.

## Materials and methods

### Animal and cell culture

Female athymic BALB/c nu/nu mice, 3–4 weeks old, obtained from HFK Bioscience (China), were maintained at the Animal Core Facility at The First Affiliated Hospital of Xinxiang Medical University, under specific pathogen-free (SPF) condition. All studies on mice were conducted in accordance with the National Institutes of Health ‘Guide for the Care and Use of Laboratory Animals’ and were approved by the Ethical Committee of The First Affiliated Hospital of Xinxiang Medical University.

### Sample collection

Ten patients with primary breast cancer, who consecutively underwent chemotherapy at Department of Gastroenterology, The First Affiliated Hospital of Xinxiang Medical University, were enrolled in the present study from January 2009 to June 2015. Informed consent for the additional core-needle biopsy and experimental use of tumor samples was obtained from all the patients, following a protocol approved by the Ethics Committee of The First Affiliated Hospital of Xinxiang Medical University.

### Sphere formation and propagation

Single-cell suspensions were obtained from colorectal cancer primary tissue samples. Colorectal cancer primary tissue samples were shipped to laboratory in cold RPMI-1640 medium with penicillin/streptomycin within 1 h of removal from patients. Surgical specimens were washed with cold PBS supplemented with high doses of penicillin/streptomycin three times, chopped with a sterile blade, and incubated in 1 mg/ml collagenase II (Sigma–Aldrich, U.S.A.) for 30 min at 37°C. The details are listed in the Supplementary material.

### Quantitative real-time RT-PCR analysis

Total RNA was isolated using TRIzol reagent (Invitrogen, U.S.A.) according to the manufacturer’s protocol. Total RNA was extracted using TRIzol reagent (Invitrogen, U.S.A.) according to the manufacturer’s protocol in the condition of low temperature. Synthesis of cDNA with reverse transcriptase was performed by PrimeScript RT Reagent Kit Perfect Real Time (TaKaRa, China). For gene expression analysis, quantitative real-time PCR analysis was done using the iCycler iQ5 real-time PCR Detection system (Bio–Rad, U.S.A.) with SYBR Green Reagent (Bio–Rad, U.S.A.). The details are listed in the Supplementary material.

### MB

MB were created in an aqueous dispersion of 2 mg/ml 1,2-distearoyl-sn-glycero-3-phosphocholine (AvantiPolar Lipids, Alabaster, AL, U.S.A.) and 1 mg/ml PEG 40 stearate (Sigma–Aldrich) using a 20-kHz sonicator (Vibra Cell; Sonics and Materials, Danbury, CT, U.S.A.) in the presence of C_3_F_8_ gas. The details are listed in the Supplementary material.

### US exposure

Three 1-MHz submersible US probes were used. A 12-mm (Fuji Ceramics, Fujinomiya, Japan) and a 30-mm diameter probe (BFC Applications, Fujisawa, Japan) were used for the *in vitro* experiments, whereas 38-mm diameter probes (Fuji Ceramics) were used for the *in vivo* experiments. Each probe was placed in the test chamber (380 mm × 250 mm × 130 mm) that was previously filled with tap water. The details are listed in the Supplementary material.

### *In vitro* quantization of calcein uptake

The ALDHA1^+^ BCSCs (5 × 10^4^ cells/well) were seeded in complete medium on to 48-well plates and incubated at 37°C in a 5% CO_2_ incubator. The medium was replaced with fresh medium containing 200 μmol/l calcein (molecular weight: 622) with and without MB (10% v/v). After US exposure for 10 s, the cells were washed with PBS, trypsinized, and collected. Twenty microliters of the supernatant was examined for the uptake of fluorescent molecules using Mx3000P software (Stratagene, CA, U.S.A.). The details are listed in the Supplementary material.

### MTT assay

ALDHA1^+^ cells, ALDHA1 cells, and differentiated adherent progeny of ALDHA1^+^ cells were seeded in 96-well plates at 2000 cells/well, respectively. Cells were then treated with increasing concentrations of DOX from 0 to 8 µg/ml for 24 h. The MTT assay (Sigma–Aldrich, U.S.A.) was conducted as given in the Supplementary material.

### Bioluminescence imaging

On days 4, 7, 9, and 11, the mice were anesthetized with isoflurane. Subsequently, they were injected intraperitoneally with luciferin (150 μg/g body weight) and placed on the *in vivo* imaging system (IVIS100; Xenogen). The bioluminescence signals were monitored at 10-s time intervals after 10-min luciferin administration. The signal intensity was quantitated as the sum of all the detected photon counts within the region of interest after subtraction of the measured background luminescence. The tumor volume was calculated according to the formula: (π/6) × (width) 2 × (length).

### Flow cytometry assay and FACS

Flow cytometry assay was done on single-cell suspensions obtained by enzymatic digestion of spheres which were derived from primary colorectal cancer samples and labeled with Alexa Fluor@488 conjugated anti-ALDHA1 antibody (Abcam, U.S.A.) by using an Epics Altra flow cytometer (Beckman Coulter, U.S.A.). FASC: spheres which were derived from primary tumor samples were dissociated into single cells by enzymatic digestion. Single-cell suspensions were washed and incubated in staining solution with 1% BSA and 2 mM EDTA with the specific antibodies at appropriate dilutions. The details are listed in the Supplementary material.

### Statistical analysis

SPSS13.0 software was used. Each experiment was performed at least three times. The data were expressed as mean ± S.D. and one-way ANOVA. An unpaired Student’s *t* test was used to determine the significant differences of all the results. Significances are ***, *P*<0.001; **, *P*<0.01; and *, *P*<0.05.

## Results

### ALDHA1^+^ spheres from primary breast cancer tissue samples and cell lines display stem cell like features

**Figure 1:**
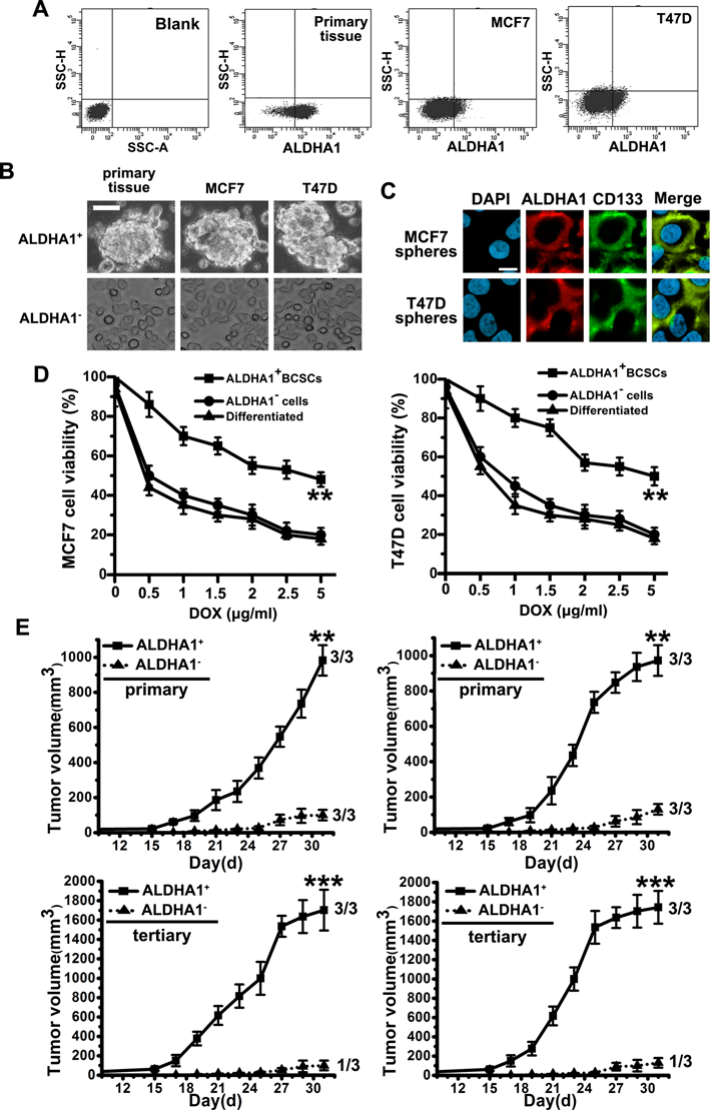
ALDHA1^+^ spheres from breast cancer primary tissue samples and cell lines display stem cell like features (**A**) Flow cytometer analysis of ALDHA1 expression in fresh breast cancer primary tissue samples and cell lines (MCF7 and T47D). (**B**) Phase-contrast images of ALDHA1^+^ sphere cells and ALDHA1^−^ cells. Scale bar, 50 μm. (**C**) Expression of CD133 in FACS-purified fraction of ALDHA1^+^ sphere progenies by immunofluorescence staining. Scale bar, 10 μm. (**D**) With the presence of DOX, cell viability of ALDHA1^+^ cells and ALDHA1^−^ cells were determined by MTT. Note: columns, mean of three individual experiments; S.D., **, *P*<0.01. (**E**) *In vivo* serial transplantation assay. A total of 10^4^ ALDHA1^+^ cells and ALDHA1^−^ cells, purified from MCF7 and T47D, were injected s.c. into nude mice. Derived tumor xenografts were dissociated to single-cell suspension and then serially reinjected in mice (10^4^ cells), generating secondary and then tertiary tumors. Tumor growth curves of primary and tertiary tumors are shown. Note: columns, mean of three individual experiments; S.D., ***, *P*<0.001; S.D., **, *P*<0.01.

CSCs are believed to be able to form spheres in serum-free cultivation. We cultured primary breast cancer tissue cells and two breast cancer cell line cells, MCF7 and T47D, to induce sphere formation in serum-free cultivation. After culturing for 2–4 weeks, even though a large number of cells died, some tumor cells grew to form spheres (results not shown). The tumor spheres were at least passaged ten times, indicating the self-renewal of these sphere cells. To identify whether ALDHA1 is the surface marker to sort BCSCs, we analyzed ten fresh tumor samples derived from a series of breast cancer patients. Flow cytometry demonstrated the presence of a variable fraction of ALDHA1^+^ cells, ranging from 0.1 to 1.7%, in nine out of ten breast cancer specimens (Figure 1A, Supplementary Table S1, Supplementary material). Then, we fractionated ALDHA1^+^ and ALDHA1^−^ cells for further study (Figure 1A). All the ALDHA1^+^ cells could form spheres in serum-free cultivation, while only a few ALDHA1^−^ cells could (Figure 1B). By staining with CD133 in ALDHA1^+^ cells, we noted that almost all the ALDHA1^+^ cells expressed CD133 (Figure 1C). Furthermore, we found that cell viability in ALDHA1^−^ cells decreased at each concentration compared with that in ALDHA1^+^ cells, with increasing concentrations of DOX from 0 to 5 µg/ml (Figure 1D). Moreover, we revealed that tumor formation of ALDHA1^+^ cells was faster and resulted in increased tumor intake compared with that observed in ALDHA1^−^ cells (Figure 1E). Meanwhile, ALDHA1^+^ cells possess higher long-term tumorigenic potential compared with the rarely observed tumors originating from the ALDHA1^−^ cells by serial transplantation assays in nude mice of cells isolated from tumor xenografts originally derived from ALDHA1^+^ or ALDHA1^−^ cells injection (Figure 1E).

### The effect of US and MB on ALDHA1^+^ BCSCs membrane permeability

**Figure 2:**
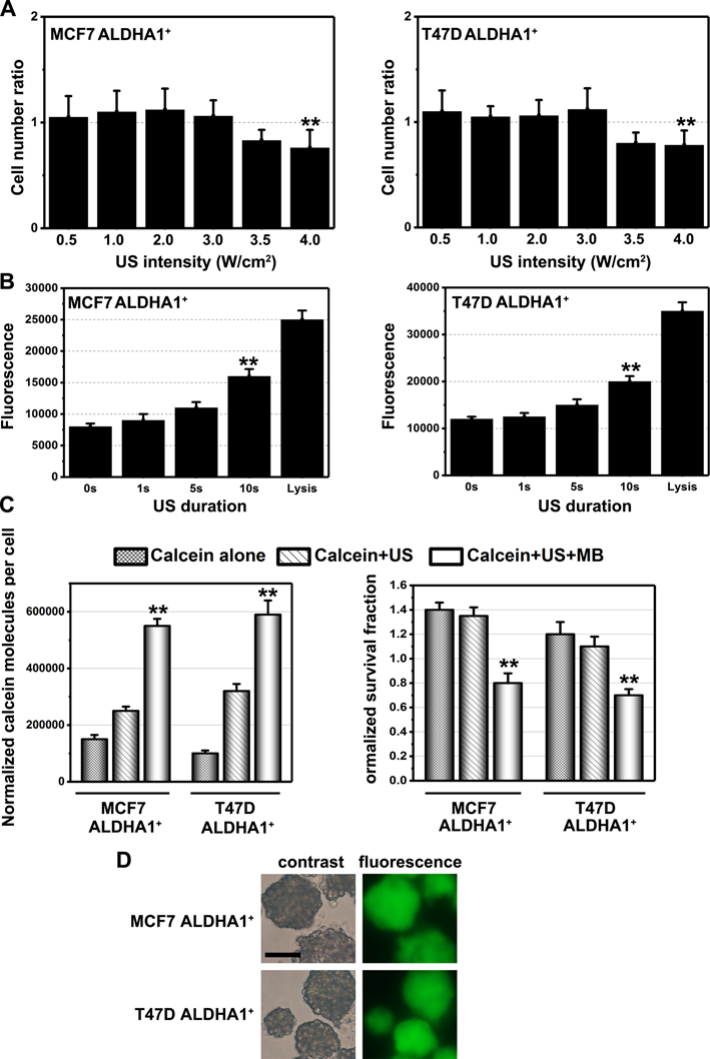
The effect of US and MB on ALDHA1^+^ BCSCs membrane permeability (**A**) Cytotoxic effect of various US intensities. Note: columns, mean of three individual experiments; S.D., **P<0.01. (**B**) Fluorescence detected using the EuTDA–Efflux assay after various amounts of US exposure. Note: columns, mean of three individual experiments; S.D., **P<0.01. (**C**) Mean fluorescence uptake under various conditions (calcein alone, calcein + US, and calcein + US + MB). Note: columns, mean of three individual experiments; S.D., **P<0.01. (**D**) Confocal microscopy showing differential interference contrast and fluorescence images exposed to US in the presence of MB. Scale bar, 50 μm.

Since, we were using a form of US not used widely, we should initially determine the optimal acoustic treatment condition that would be the most effective to enhance ALDHA1^+^ BCSCs membrane permeability while avoiding adverse aspects such as hyperthermia. Cultured ALDHA1^+^ BCSCs were treated by US in several conditions. When the US intensity was set at 1.0–4.0 W/cm^2^, there was no significant difference in cell counts compared with control cells (Figure 2A). Then, the results of the EuTDA–Efflux assay in Figure 2B compared with the US treatment time. The fluorescence observed was plotted for each duration of ultrasonic irradiation, with fluorescence being directly proportional to the amount of EuTDA effluxed from cells. In general, the fluorescence increases as the duration of ultrasonic exposure increases with the greatest amount of fluorescence occurring in the cells treated with lysis buffer (positive control) and the least amount of fluorescence occurring in the cells treated with 0 s of ultrasonic irradiation (negative control). The fluorescences observed after 10 s of US treatment were much greater than the fluorescence observed with no US exposure (Figure 2B).

To explore the effect of MB, we employed calcein to serve as the marker to detect the uptake of DOX into ALDHA1^+^ BCSCs. The exposure of cells to US in the presence of MB resulted in the delivery of 10^6^–10 ^7^ calcein molecules per cell (Figure 2C). This represents a significant increase in the uptake of fluorescent molecules compared with calcein alone or calcein + US. Moreover, confocal fluorescence microscopic analysis was used to confirm that the calcein molecules actually entered the cytoplasm (Figure 2D).

### US-MB treatment makes ALDHA1^+^ BCSCs sensitive to DOX *in vitro* by apoptosis

**Figure 3:**
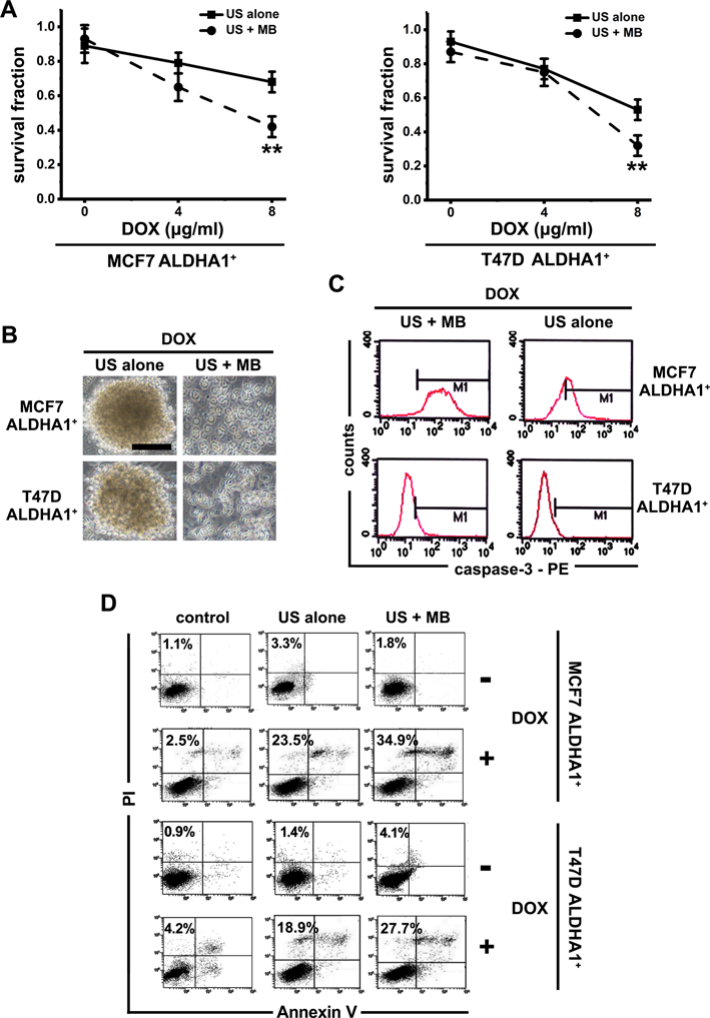
US-MB treatment makes ALDHA1^+^ BCSCs sensitive to DOX *in vitro* (**A**) With the presence of DOX, cell viability of ALDHA1^+^ cells with different treatments were determined by MTT. Note: columns, mean of three individual experiments; S.D., **, *P*<0.01. (**B**) Phase-contrast images of ALDHA1^+^ sphere with different treatments. Scale bar, 50 μm. (**C**) The caspase-3 activity in ALDHA1^+^ BCSCs with different treatment was measured by flow cytometry. (**D**) With or without DOX, the percentage of Annexin V^+^ PI^+^/Annexin V^+^ PI^−^ cells in different group was measured by flow cytometry.

Next, the cytotoxicity of various doses of DOX in the presence of US with and without MB was tested on ALDHA1^+^ BCSCs. A marked increase in DOX toxicity was observed under the US-MB conditions, whereas US alone did not significantly affect cell survival with various DOX concentrations (Figure 3A). MTT assay also showed that US-MB achieved a very limited loss of cell viability (Figure 3A). However, the survival fraction rate due to MB alone was not investigated as it was found that MB alone did not contribute to cell viability (results not shown). Meanwhile, under the US-MB conditions, ALDHA1^+^ BCSCs treated with DOX could not form compact spheres, while the group at the absence of US-MB could form spheres at different degrees (Figure 3B).

Additionally, DOX is well known to induce apoptosis; thus, we confirmed the involvement of apoptosis in mediating cytotoxicity in response to DOX. US + MB + DOX activated caspase-3 expression in ALDHA1^+^ BCSCs as compared with that in other groups (Figure 3C). Moreover, Annexin V/PI staining assay was used in each group. We found that ALDHA1^+^ BCSCs treated with US + MB + DOX showed increased percentage of Annexin V^+^PI^+^/Annexin V^+^PI^−^ cells as compared with that in the other group (Figure 3D). Taken together, these data demonstrated that the DOX + US + MB combination decreased ALDHA1^+^ BCSCs cell viability and that this reduction in cell survival was associated with increased induction of apoptosis.

### *In vivo* therapeutic effects of US-MB treatment

**Figure 4:**
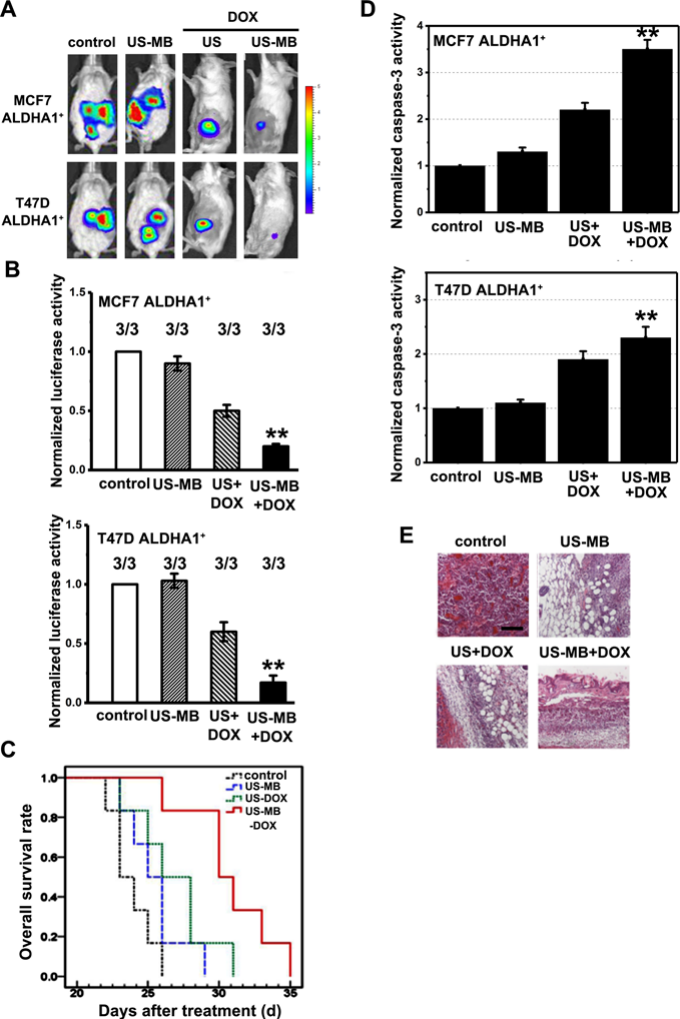
*In vivo* therapeutic effects of US-MB treatment (**A**) Bioluminescence imaging and luciferase activity of mice with the treatment at US intensity: 3.0 W/cm^2^; duty cycle: 20%; number of pulses: 200; pulse repletion frequency: 1000 Hz; and exposure time: 60 s. (**B**) The luciferase activity normalized with that of control at day 11, where the concentration of DOX was 1.25 μg/g of body weight (*n*=3). (**C**) Kaplan–Meier survival curves for different mice tumor group showed a statistical difference in different treatment. (**D**) The caspase-3 level in different mice tumor group was measured by quantitative real-time RT-PCR analysis (qPCR). Note: columns, mean of three individual experiments; S.D., **, *P*<0.01. (**E**) Representative histopathological changes in the control, US + MB alone, US + DOX, and US + MB + DOX groups. Scale bar, 10 μm.

We, then, investigated the antitumor effects of US + MB in the presence of DOX *in vivo*. To determinate the concentration of DOX, we tested the two different DOX concentrations (0.5 or 1.25 μg/g) on days 7, 9, and 11 (results not shown). The antitumor effects of the US + MB + DOX were recognized after day 7 in both groups. On day 11, a significant reduction was observed in the DOX + US + MB group at 1.25 μg/g (Figure 4A). Figure 4B showed antitumor effects for different conditions (US + MB, DOX + US, and DOX + US + MB) at day 11. Furthermore, the DOX + US + MB group showed a prolonged median survival compared with the DOX + US group or the US + MB group (Figure 4C). Meanwhile, we found that xenografts in DOX + US + MB group expressed increased caspase-3 (Figure 4D). In addition, H&E staining revealed a reduction in hyperemia, necrosis, and the number of small vessels decreased in the US-MB + DOX group compared with the other three groups (Figure 4E).

### US-MB-DOX treatment decreases the ABCG2 expression in ALDHA1^+^ BCSCs

**Figure 5:**
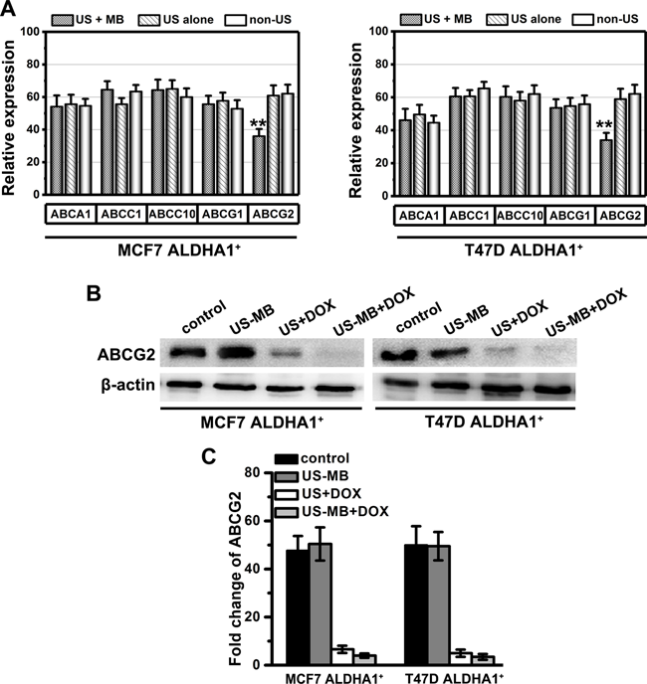
US-MB-DOX treatment **decreases** the ABCG2 expression in ALDHA1^+^ BCSCs (**A**) The ABC transporter expression level in different group *in vitro* was measured by quantitative real-time RT-PCR analysis (qPCR). Note: columns, mean of three individual experiments; S.D., **, *P*<0.01. (**B**) Western blotting showed that ABCG2 protein level in different mice tumor tissues and (**C**) related gray coefficient ratio for every band of Western blot. β-actin was used as a normalization control.

So far, there are a number of mechanisms that may contribute to chemoresistance of cancer cells. Amongst these, decreased intracellular drug accumulation caused by drug efflux pumps in the cell membrane is one of the most common mechanisms. These membrane efflux pumps belong to the superfamily of ATP-binding cassette (ABC) transporter proteins and contribute to drug resistance via ATP-dependent pathways [11]. Here, we found that, *in vitro*, the expression of ABCG2 was reduced significantly in ALDHA1^+^ BCSCs in the presence of US-MB compared with ALDHA1^+^ BCSCs in the presence of US alone (Figure 5A). Meanwhile, *in vivo*, ABCG2 was found decreased in mice tumor tissues in the presence of US-MB compared with the other group (Figure 5B). These data suggested that decreased ABCG2 level might be one of the mechanisms by which US-MB medicated DOX treatment.

## Discussion

Therapeutic US, especially low-intensity US, has gained increasing attention in recent years [12]. In general, US can cause both physical effects (such as heat, cavitation, and mechanical force) and biochemical effects (such as reactive oxygen species) to affect tumor cell damage [13]. MB can be intentionally ruptured for cavitation effect which is caused by localized US energy [14]. Violent microstreaming, shock wave, or efflux can be produced during the event of collapse of MB, which can cause transient holes in the plasma membrane to increase permeability of cell membrane. This specific drug delivery method potentially serves to minimize toxic side effects, lower the required dosage amounts, and reduce the cost of patients [15].

BCSCs are reported as the major source of tumor recurrence after radiation or chemotherapy [3]. It was reported that the percentage of BCSCs following chemotherapy is increased as compared with that before therapy [6]. Residual BCSCs which chemotherapy fails to kill will eventually lead to subsequent disease recurrence of breast cancer patients. Nevertheless, there are still not any strategies helping to remove BCSCs by enhancing their sensitivity to chemotherapy drugs. In the present study, we demonstrated that US-MB treatment makes ALDHA1^+^ BCSCs sensitive to DOX by down-regulating *ABCG2* gene expression. We, first, indicated that ALDHA1^+^ spheres from primary breast cancer tissue samples and cell lines display stem cell like features, including strong ability of sphere formation *in vitro*, chemoresistance, and tumoregenesis *in vivo*. ALDHA1^+^ cells possessed the long-term tumorigenic potential by using serial transplantation assays in mice of cells isolated from tumor xenografts originally derived from ALDHA1^+^ cells injection, indicating the existence of a precise hierarchical model for the formation of lung cancer tissue, based on the generation of a vast cell progeny by a small number of self-renewing undifferentiated cells. It is likely that ALDHA1^+^ cells comprise two populations of cells with similar phenotype, but different potential: a tumorigenic population of stem cell like cells able to self-renew and a non-tumorigenic population of progenitor/precursor cells. We also found that ALDHA1^+^ BCSCs expressed high level CD133 which is the hallmark of CSCs [3]. Furthermore, we showed the presence of a variable fraction of ALDHA1, ranging from 0.1 to 2.2%, in nine out of ten breast cancer specimens.

Most studies focussed on the low intense US might have effects on adherent cancer cells. Previously, we tried to use low intense US (1.0–3.0 W/cm^2^) for BCSCs derived from MCF7 and T47D. Nevertheless, the membrane permeability of BCSCs was not good enough for DOX entering, which was also tested by fluorescence and calcein assay, as shown in Figure 2A. Therefore, the different intense US was first to be explored on BCSCs in the present study. Here, we found the optimal acoustic treatment intensity for ALDHA1^+^ BCSCs *in vitro*. Calcein, which has a molecular weight of 622 (calculated stokes radius of 0.68), was used as a fluorescent marker to evaluate small molecule entry in cancer cells upon US-MB stimulation. As the molecular weight of DOX is smaller than calcein, so calcein can be considered to represent a realistic marker of DOX entry into ALDHA1^+^ BCSCs. Furthermore, we showed that US-MB treatment made ALDHA1^+^ BCSCs sensitive to DOX with the decreasing ability of sphere formation and the increase in caspase-3 activity. Nevertheless, whether the activation of caspase-3 is induced by US needs further study, as we showed that US alone did not contribute to the survival fraction and subsequent apoptosis induction. We also demonstrated that US-MB treatment had better efficiency in enhancing membrane permeability compared with US alone treatment. As for *in vivo*, DOX + US + MB group showed a better antitumor result and a prolonged median survival compared with other groups.

So far, there are a number of mechanisms that may contribute to chemoresistance of cancer cells. Amongst these, decreased intracellular drug accumulation caused by drug efflux pumps in the cell membrane is one of the most common. Here, significantly, we found that the expression of ABCG2 was reduced in ALDHA1^+^ BCSCs in the presence of US-MB compared with that in the presence of US alone. Additionally, in *in vivo* assays, we also found that the decreased expression of ABCG2 in DOX + US + MB group in line with the results of *in vitro* experiments, which suggested that decreased ABCG2 level might be one of the mechanisms by which US-MB medicated DOX treatment. Nevertheless, the exact relationship or mechanisms between US-MB medicated DOX treatment and ABC transporters needs further study.

In conclusion, we showed a new US-MB treatment condition on ALDHA1^+^ BCSCs, for the first time. We demonstrated the efficacy of combined treatment on human BCSCs *in vitro* and *in vivo* with using DOX and US-MB: the combined treatment with much reduced drug dosage significantly suppressed the stem cell like features of BCSCs and induced BCSCs apoptosis. Furthermore, we suggested that decreased ABCG2 level might be one of the mechanisms by which US-MB medicated DOX treatment. All in all, this new US-MB treatment condition has clinical potential in breast cancer therapy by targetting BCSCs.

## Supporting information

**Table S1:** Expression of ALDH1A1 in primary breast cancer samples

## Abbreviations

ABC: ATP-binding cassette
RT: real-time
H&E: hematoxylin and eeosin staining
BCSC: breast cancer stem cell like cell
CSC: cancer stem cell like cell
DOX: doxorubicin
MB: microbubble
US: ultrasound

## References

[1] DeSantis C., Ma J., Bryan L. and Jemal A. (2014) Breast cancer statistics, 2013. CA Cancer J. Clin. 64, 52–62 24114568 10.3322/caac.21203

[2] Su J., Wu S., Wu H., Li L. and Guo T. (2016) CD44 is functionally crucial for driving lung cancer stem cells metastasis through Wnt/beta-catenin-FoxM1-Twist signaling. Mol. Carcinog. 55, 1962–1973 26621583 10.1002/mc.22443

[3] Bertolini G., Roz L., Perego P., Tortoreto M., Fontanella E., Gatti L. (2009) Highly tumorigenic lung cancer CD133+ cells display stem-like features and are spared by cisplatin treatment. Proc. Natl. Acad. Sci. U.S.A. 106, 16281–16286 19805294 10.1073/pnas.0905653106 PMC2741477

[4] Lapidot T., Sirard C., Vormoor J., Murdoch B., Hoang T., Caceres-Cortes J. (1994) A cell initiating human acute myeloid leukaemia after transplantation into SCID mice. Nature 367, 645–648 7509044 10.1038/367645a0

[5] Ricci-Vitiani L., Lombardi D.G., Pilozzi E., Biffoni M., Todaro M., Peschle C. (2007) Identification and expansion of human colon-cancer-initiating cells. Nature 445, 111–115 17122771 10.1038/nature05384

[6] Lv Y., Wang T., Fan J., Zhang Z., Zhang J., Xu C. (2017) The effects and mechanisms of SLC34A2 on maintaining stem cell-like phenotypes in CD147^+^ breast cancer stem cells. Tumour Biol. 39, 1010428317695927 28381172 10.1177/1010428317695927

[7] Schlicher R.K., Radhakrishna H., Tolentino T.P., Apkarian R.P., Zarnitsyn V. and Prausnitz M.R. (2006) Mechanism of intracellular delivery by acoustic cavitation. Ultrasound Med. Biol. 32, 915–924 16785013 10.1016/j.ultrasmedbio.2006.02.1416

[8] Stenzel M. and Mentzel H.J. (2014) Ultrasound elastography and contrast-enhanced ultrasound in infants, children and adolescents. Eur. J. Radiol. 83, 1560–1569 25022978 10.1016/j.ejrad.2014.06.007

[9] Taniyama Y., Tachibana K., Hiraoka K., Namba T., Yamasaki K., Hashiya N. (2002) Local delivery of plasmid DNA into rat carotid artery using ultrasound. Circulation 105, 1233–1239 11889019 10.1161/hc1002.105228

[10] Carvalho C., Santos R.X., Cardoso S., Correia S., Oliveira P.J., Santos M.S. (2009) Doxorubicin: the good, the bad and the ugly effect. Curr. Med. Chem. 16, 3267–3285 19548866 10.2174/092986709788803312

[11] Szakacs G., Paterson J.K., Ludwig J.A., Booth-Genthe C. and Gottesman M.M. (2006) Targeting multidrug resistance in cancer. Nat. Rev. Drug Discov. 5, 219–234 16518375 10.1038/nrd1984

[12] McHale A.P., Callan J.F., Nomikou N., Fowley C. and Callan B. (2016) Sonodynamic therapy: concept, mechanism and application to cancer treatment. Adv. Exp. Med. Biol. 880, 429–450 26486350 10.1007/978-3-319-22536-4_22

[13] Yu T., Wang Z. and Mason T.J. (2004) A review of research into the uses of low level ultrasound in cancer therapy. Ultrason. Sonochem. 11, 95–103 15030786 10.1016/S1350-4177(03)00157-3

[14] Heller L.C. and Heller R. (2006) *In vivo* electroporation for gene therapy. Hum. Gene Ther. 17, 890–897 16972757 10.1089/hum.2006.17.890

[15] Rapoport N.Y., Kennedy A.M., Shea J.E., Scaife C.L. and Nam K.H. (2009) Controlled and targeted tumor chemotherapy by ultrasound-activated nanoemulsions/microbubbles. J. Control. Release 138, 268–276 19477208 10.1016/j.jconrel.2009.05.026 PMC2746980

