# Supplementary material for: Ultrasound reverses chemoresistance in breast cancer stem cell like cells by altering ABCG2 expression
Source: Biosci Rep. 2017 Nov 9;37(6):BSR20171137. doi: 10.1042/BSR20171137 (PMC5678030; doi:10.1042/BSR20171137)
Supplement: Supplementary file 1 [file bsr20171137_Supp1.pdf]

Supplemental Information

MATERIALS AND METHODS

Animal and Cell culture

Female athymic BALB/c nu/nu mice, 3-4 week-old, obtained from HFK Bioscience (China), were maintained at the Animal Core Facility at The First Affiliated Hospital of Xinxiang Medical University, under specific pathogen-free (SPF) condition. All studies on mice were conducted in accordance with the National Institutes of Health 'Guide for the Care and Use of Laboratory Animals' and were approved by the ethical committee of The First Affiliated Hospital of Xinxiang Medical University.

Sample Collection

10 patients with primary breast cancer, who consecutively underwent chemotherapy at Department of Gastroenterology, The First Affiliated Hospital of Xinxiang Medical University, were enrolled into this study from January 2009 to June 2015. Informed consent for the additional core-needle biopsy and experimental use of tumor samples was obtained from all patients, following a protocol approved by the Ethics Committee of The First Affiliated Hospital of Xinxiang Medical University.

Sphere formation and propagation

Single-cell suspensions were obtained from colorectal cancer primary tissue samples. Colorectal cancer primary tissue samples were shipped to laboratory in cold RPMI-1640 medium with penicillin/streptomycin within 1 hour of removal from patients. Surgical specimens were washed with cold PBS supplemented with high doses of penicillin/streptomycin three times, chopped with a sterile blade, and incubated in 1 mg/ml collagenase II (Sigma-Aldrich, USA) for 30 min at 37℃. After incubation, the suspensions were repeatedly triturated, passed through 70 μm cell strainers (BD Falcon, USA). Recovered cells were cultured at clonal density in serum-free medium containing N2 (100X) supplementary (Gibco-Invitrogen, USA), 0.4% BSA (Gibco-Invitrogen, USA), 100U/ml penicillin, 1mg/ml streptomycin in DMEM–F12 medium (Gibco-Invitrogen, USA) and supplemented with 20 mg/ml EGF, 20 mg/ml IGF and 20 mg/ml bFGF. Flasks non-treated for tissue culture were used to reduce cell adherence and support growth as undifferentiated tumor spheres.

Quantitative Real-time RT-PCR Analysis (qPCR)

Total RNA was isolated by using Trizol reagent (Invitrogen, USA) according to the manufacturer's protocol. Total RNA was extracted using Trizol reagent (Invitrogen, USA) according to the manufacturer's protocol in the condition of low temperature. Synthesis of cDNA with reverse transcriptase was performed by PrimeScript RT reagent Kit Perfect Real Time (TaKaRa, China). For genes expression analysis, quantitative real-time PCR analysis was done using the iCycler iQ5 real-time PCR Detection system (Bio-Rad, USA) with SYBR Green Reagents (Bio-Rad, USA). Β-actin was amplified as an internal control. Comparative gene expression analysis was performed using the 2 -ΔΔCt method with normalization to the level of internal control β-actin.

Microbubbles

MB were created in an aqueous dispersion of 2mg/mL 1,2-distearoyl-sn-glycero-3-phosphocholine (AvantiPolar Lipids, Alabaster, AL, USA) and 1

mg/mL polyethyleneglycol 40 stearate (Sigma-Aldrich) using a 20-kHz sonicator (Vibra Cell; Sonics and Materials, Danbury, CT, USA) in the presence of C_3F_8 gas. The lipid molecules that formed components of the MB surface were confirmed by staining the molecules with 3 μ mol/L FM1-43 (excitation 479 nm, emission 598 nm; Molecular Probes, Eugene, OR, USA) and observing them under an inverted microscope (IX81; Olympus, Tokyo, Japan). The peak diameter and the zeta potential of the MB were determined to be 1272 ± 163 nm (n = 7) and –4.1 ± 0.85 mV (n = 3), respectively, by using a laser diffraction particle size analyzer (particle range 0.6 nm–7 μ m; ELSZ-2; Otsuka Electronics, Osaka, Japan).

Ultrasound exposure

Three 1-MHz submersible US probes were used. A 12-mm (Fuji Ceramics, Fujinomiya, Japan) and a 30-mm diameter probe (BFC Applications, Fujisawa, Japan) were used for the in vitro experiments, whereas 38-mm diameter probes (Fuji Ceramics) were used for the in vivo experiments. Each probe was placed in the test chamber (380 mm × 250 mm × 130 mm) that was previously filled with tap water. The positive and negative peak values of the pressures were the same. 4 W/cm^2, were used in the in vitro experiments. The duty cycle was 50%, the number of pulses was 2000, the pulse repletion frequency was 250 Hz, and the exposure time was 10 s. For the in vivo experiments, the intensity was 5 W/cm^2, the duty cycle was 20%, the number of pulses was 200; the pulse repletion frequency was 1000 Hz, and the exposure time was 60 s. The intensity was defined as the average rate of flow of energy through a unit area placed normal to the direction of propagation.

In vitro quantization of calcein uptake

The $ALDHA1^+$ BCSCs (5 × 10^4 cells/well) were seeded in complete medium onto 48-well plates and incubated at 37 ° C in a 5% CO_2 incubator. The medium was replaced with fresh medium containing 200 μ mol/L calcein (molecular weight 622) with and without MB (10% v/v). After US exposure for 10 s, the cells were washed with phosphate-buffered saline (PBS), trypsinized, and collected. Thereafter, the cells were washed three times and transferred to a 1.5-mL conical tube in which they were pelleted. The pellets were lysed in 200 μ L reporter lysis buffer (Promega, Madison, WI, USA) and subsequently frozen at –80 ° C for 15 min. The cells were thawed on ice. Each cell lysate was centrifuged at 12 000g for 2 min to pellet the cell debris. 20μL of the supernatant was examined for the uptake of fluorescent molecules using Mx3000P software (Stratagene, CA, USA).

MTT assay

$ALDHA1^+$ cells, ALDHA1 cells, and differentiated adherent progeny of $ALDHA1^+$ cells were seeded in 96-well plates at 2000 cells/well, respectively. Cells were then treated with increasing concentrations of doxorubicin from 0 to 8 μg/mL for 24 hours. The MTT assay (Sigma Aldrich, USA) was used to determine relative cell growth every 24 h for cell growth curves. 20μl of 5mg/ml MTT was added to the media for 4h incubation at 37℃. Following removal of the culture medium, the remaining crystals were dissolved in 150μl DMSO (Sigma Aldrich, USA). The curve of growth was drawn with the absorbance (A) measured spectrophotometrically in a microplate reader (Bio-Rad, USA) at a wavelength of 490 nm.

Bioluminescence imaging

On days 4, 7, 9, and 11, the mice were anesthetized with isoflurane. Subsequently, they were injected intraperitoneally with luciferin (150 μ g/g bodyweight) and placed on the in vivo imaging system (IVIS100; Xenogen). The bioluminescence signals were monitored at 10-s time intervals

after 10 min luciferin administration. The signal intensity was quantified as the sum of all detected photon counts within the region of interest after subtraction of the measured background luminescence. The tumor volume was calculated according to the formula (π/6) × (width) 2 × (length).

Flow cytometry assay and fluorescence activated cell sorting (FACS)

Flow cytometry assay was done on single-cell suspensions obtained by enzymatic digestion of spheres which were derived from primary colorectal cancer samples and labelled with Alexa fluor@488 conjugated anti-ALDHA1 antibody (Abcam, USA) by using an Epics Altra flow cytometer (Beckman Coulter, USA). FASC: spheres which were derived from primary tumor samples were dissociated into single cells by enzymatic digestion. Single-cell suspensions were washed and incubated in staining solution 1% BSA and 2mM EDTA with the specific antibodies at appropriate dilutions. 10^6 cells were incubated with appropriate concentration of antibodies. Cells were stained with Alexa fluor@488 conjugated anti-ALDHA1 antibody (Abcam, USA) staining, along with appropriate negative controls and single-color positive controls. Sorted cells were cultured in serum-free DMEM/F12 medium with supplementary as the same as all above at 37°C in a 5% CO_2 humidified incubator.

Quantitative real-time RT-PCR analysis (qPCR)

Total RNA was extracted using TRIzol (Invitrogen, USA) and treated with RNase-free DNase (Qiagen, USA). Mature miRNA expression analysis was conducted using a TaqMan MicroRNA Assays (Applied Biosystems, USA). qPCR was performed using a SYBR Green Reagents (Bio-Rad, USA) on the iQ5 Real-Time PCR Detection System (Bio-Rad, USA). The relative expression levels of the mRNAs in cells were normalised to β-actin and those in the serum and exosomes were normalised to the insert control according to the manufacturer’s protocol.

Western blot

Protein extracts were resolved through 8% to 12% SDS-PAGE; transferred to nitrocellulose membranes; and probed with mouse monoclonal antibody against ABCG2 (Abcam, USA), ABCA1 (Abcam, USA) or β-actin (Proteintech, USA); probed with peroxidase-conjugated secondary antibody (Proteintech, USA). The membranes were washed and incubated with a horseradish peroxidase (HRP)-conjugated secondary antibody. Protein expression was detected and quantified using the ODYSSEY Infrared Imaging System (Li-COR Biosciences, USA).

Statistical Analysis

SPSS13.0 software was used. Each experiment was performed at least three times. The data were expressed as mean ± SD and one-way ANOVA. An unpaired Student's t-test were used to determine the significant differences of all the results. Significances are, ***, $p < 0.001$; **, $p < 0.01$; *, $p<0.05$.

Table S1. Expression of ALDH1A1 in primary breast cancer samples

Case	Age	TNM	Clinical stage	Percentage of $ALDH1A1^+$cells	Sphere formation
1	46	T2N1M0	IIA	0.2%	Yes
2	55	T2N0M0	IB	0.1%	Yes
3	44	T1N1M0	IIA	1.1%	Yes

4	31	T2N1M0	IIA	0.7%	Yes
5	57	T2N2M0	IIB	1.1%	Yes
6	46	T2N3M0	IIIB	0.8%	No
7	58	T3N3M1	IV	0.7%	Yes
8	40	T2N2M0	IIIA	1.3%	Yes
9	56	T2N0M0	IB	1.9%	Yes
10	52	T2N0M0	IB	1.2%	Yes
